# Estimating the relative proportions of SARS-CoV-2 haplotypes from wastewater samples

**DOI:** 10.1016/j.crmeth.2022.100313

**Published:** 2022-09-20

**Authors:** Lenore Pipes, Zihao Chen, Svetlana Afanaseva, Rasmus Nielsen

**Affiliations:** 1 Department of Integrative Biology, University of California-Berkeley, 4098 Valley Life Sciences Building, Berkeley, CA 94720, USA; 2 School of Mathematical Sciences, Peking University, Beijing 100871, China; 3 GLOBE Institute, University of Copenhagen, Copenhagen, Denmark

**Keywords:** SARS-CoV-2, wastewater surveillance, COVID-19, wastewater-based epidemiology, expectation maximization, imputation

## Abstract

Wastewater surveillance has become essential for monitoring the spread of severe acute respiratory syndrome coronavirus 2 (SARS-CoV-2). The quantification of SARS-CoV-2 RNA in wastewater correlates with the coronavirus disease 2019 (COVID-19) caseload in a community. However, estimating the proportions of different SARS-CoV-2 haplotypes has remained technically difficult. We present a phylogenetic imputation method for improving the SARS-CoV-2 reference database and a method for estimating the relative proportions of SARS-CoV-2 haplotypes from wastewater samples. The phylogenetic imputation method uses the global SARS-CoV-2 phylogeny and imputes based on the maximum of the posterior probability of each nucleotide. We show that the imputation method has error rates comparable to, or lower than, typical sequencing error rates, which substantially improves the reference database and allows for accurate inferences of haplotype composition. Our method for estimating relative proportions of haplotypes uses an initial step to remove unlikely haplotypes and an expectation maximization (EM) algorithm for obtaining maximum likelihood estimates of the proportions of different haplotypes in a sample. Using simulations with a reference database of >3 million SARS-CoV-2 genomes, we show that the estimated proportions reflect the true proportions given sufficiently high sequencing depth.

## Highlights

• Accurately imputes missing nucleotides using the global SARS-CoV-2 phylogeny
• Accurately estimates true proportions of SARS-CoV-2 haplotypes in wastewater samples
• Enables the use of complex shotgun sequencing datasets for SARS-CoV-2 surveillance

## Motivation

Quantification of haplotype composition in wastewater samples has the potential to become a cost-effective method to identify changes in viral community composition as SARS-CoV-2 becomes an endemic virus. We sought to develop a computational method that accurately estimates proportions of SARS-CoV-2 haplotypes from wastewater samples.

## Graphical abstract

**Figure:**
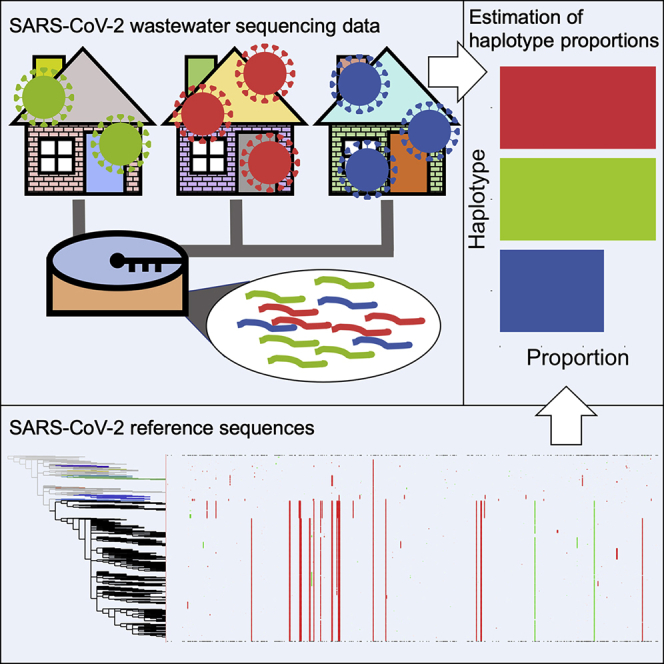

## Teaser

Pipes et al. present a method for estimating the proportion of different SARS-CoV-2 haplotypes from shotgun sequencing of wastewater samples, allowing researchers to obtain accurate results in real time.

## Introduction

The ongoing pandemic of coronavirus disease 2019 (COVID-19) caused by severe acute respiratory syndrome coronavirus 2 (SARS-CoV-2) continues to be the world’s worst public health emergency in the last century. There is an emerging need to identify the initiation of outbreaks, distribution, and changing trends of COVID-19 in near real time (Korber et al., 2020; Rockett et al., 2020). Wastewater-based epidemiology (WBE) has become an effective monitoring strategy for early detection of SARS-CoV-2 in communities as well as being an important method for informing public health interventions aimed at containing and mitigating COVID-19 outbreaks (Ahmed et al., 2020). WBE for SARS-CoV-2 can detect the virus excreted by both symptomatic and asymptomatic individuals alike, thus making it an effective approach for modeling the disease signature of entire communities. WBE data also strongly correlate with the COVID-19 case rates in a community (Medema et al., 2020; Farkas et al., 2020). Currently, most analyses of WBE data for SARS-CoV-2 focus on identifying presence/absence as well as quantifying the abundance of the virus (Kumar et al., 2020; Crits-Christoph et al., 2021; Wu et al., 2020; Medema et al., 2020). However, identifying and profiling multiple SARS-CoV-2 genotypes in a single sample can provide additional information for understanding the dynamics and transmission of certain strains. The alarming continued emergence of novel variants such as the Delta variant B.1.617.2 and the Omicron variant B.1.1.529 underscores the urgency and need for quantification of the abundance of different viral strains across communities. Unfortunately, it is difficult to precisely quantify the proportions of different haplotypes of a virus in an environmental sample, such as wastewater, using standard sequencing technologies given the low quality and highly uneven depth of sequencing data. Adding to these challenges is that many haplotypes are nearly identical, differing by only one or a few mutations across approximately ∼30,000 nucleotides. With millions of possible candidate haplotypes, the combinatorial challenge of identifying the correct haplotype is large, particularly when haplotypes are not identified by individual diagnostic mutations but rather by sets of mutations that jointly help distinguish the haplotypes from each other. Nonetheless, quantification of haplotype composition in WBE data has the potential to become a cost-effective method to identify changes in viral community composition as SARS-CoV-2 becomes an endemic virus. We present a method for estimating the proportion of different SARS-CoV-2 haplotypes from shotgun sequencing of wastewater samples, allowing researchers to obtain results in real time. The method is based on an initial filtering step, phylogenetic imputation of missing nucleotides, and an expectation maximization (EM) algorithm for obtaining maximum likelihood estimates of the proportions of different haplotypes in the sample. Using simulations, we show that the estimated proportions are close to the true proportions and that the phylogenetic imputation is highly accurate and improves the reference haplotypes. We also apply this method to wastewater samples collected across the San Francisco Bay Area and from San Diego (CA, USA).

## Results

### Imputation

**Figure 1:**
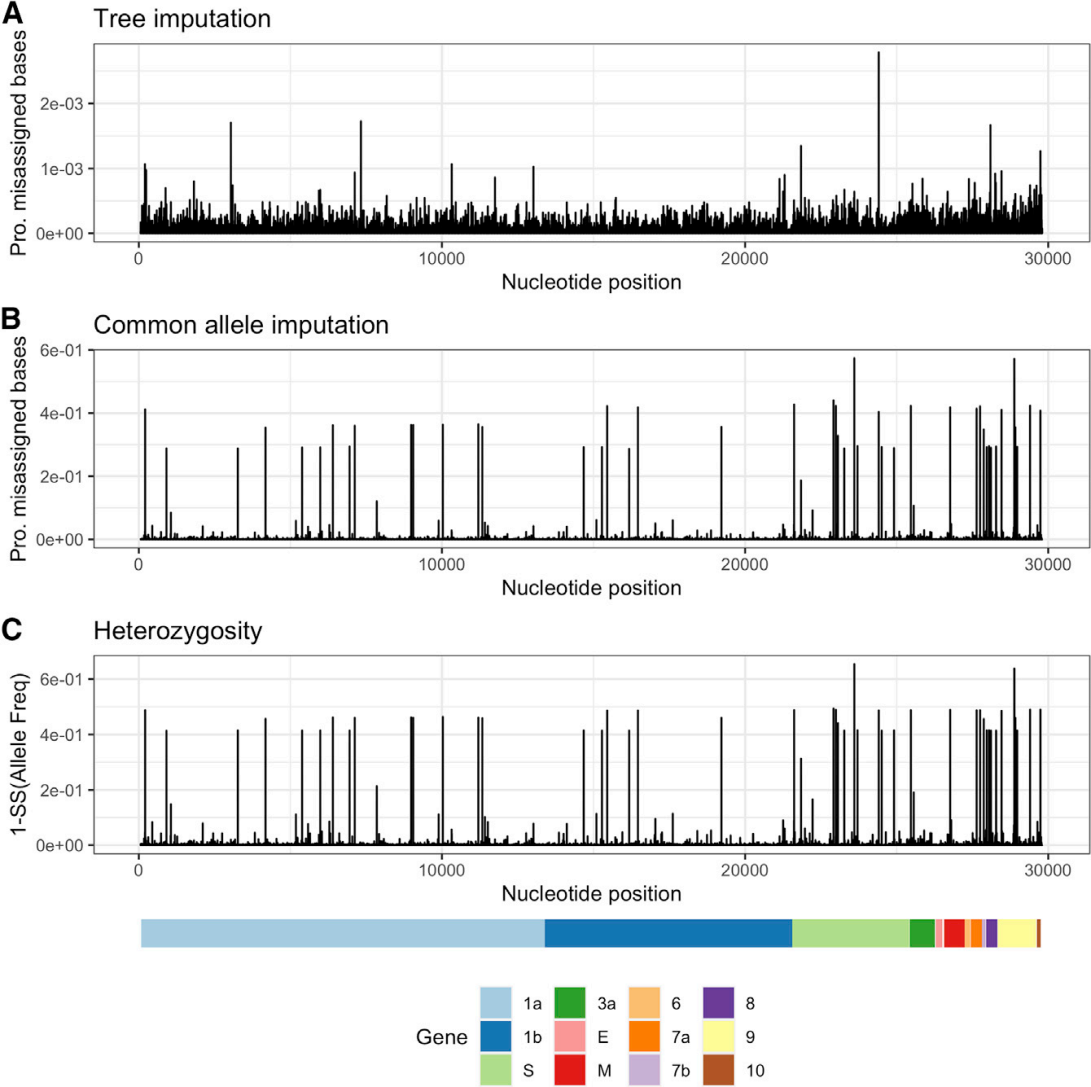
Proportion of misassigned bases for two imputation methods and heterozygosity along SARS-CoV-2 Proportion of misassigned bases along SARS-CoV-2 using the *tree imputation* method (A) and the *common allele imputation* method (B) against heterozygosity (C) using 3,117,131 SARS-CoV-2 genomes. Notice the difference in the scaling of the y axis of (A) and (B).

Many SARS-CoV-2 sequences submitted to public databases contain missing data (i.e., bases that are not coded as A, G, C, or T). This poses a problem when estimating the fraction of different SARS-CoV-2 haplotypes, as haplotypes with a high proportion of missing data, on average, will contain fewer nucleotide differences when compared with sequencing reads. We solve this problem using an imputation approach, thereby allowing for a like-to-like comparison of reads against all reference haplotypes. This method is in a spirit similar to imputation approaches used in human genetics (e.g., Marchini and Howie, 2010), although as we will show that, due to the strong phylogenetic structure in the SARS-CoV-2 data, imputation is much more accurate than usually observed in diploid organisms. The method is based on calculating the posterior probability of each nucleotide in the leaf node of a phylogenetic tree and imputing based on the maximum posterior probability (see STAR Methods). We compare the method (*tree imputation*) with a naive imputation approach based on simply replacing missing nucleotides with the most frequent nucleotide observed in the alignment in that position (*common allele imputation*). We evaluate the methods by first removing sequenced nucleotides in a real dataset of 3,117,131 SARS-CoV-2 sequences and then re-imputing them using either *tree imputation* or *common allele imputation*. For the vast majority of sites, *tree imputation* has an error rate of <5 × 10^−4^, although a few sites have imputation errors between 10^−3^ and 3 × 10^−3^ (Figure 1). The imputation error can be substantially higher for the naive *common allele imputation* method, with many sites showing error rates >0.02 (Figure 1B). These are sites with high heterozygosity (Figure 1C), where substituting with the most common allele leads to high error rates. While the error rates for the *common allele imputation* method are naturally predicted by the heterozygosity, the pattern is somewhat different for the *tree imputation* method. The sites with the highest imputation error are not the sites with highest heterozygosity, suggesting a high degree of homoplasy in these sites not directly predictable by the heterozygosity. These may be sites that switch allelic state often, i.e., have high mutation rates, but where the minor allele never increases substantially in frequency due to selection. An alternative explanation is sequencing errors. In fact, the site with the highest amount of apparent imputation error (position 24,410) is a site known to have a high proportion of sequencing errors (https://github.com/W-L/ProblematicSites_SARS-CoV2). It is located in a primer binding site where sequences containing the non-reference allele, A, often erroneously are assigned back to the reference allele, G, as a result of failed primer trimming during consensus building (https://github.com/W-L/ProblematicSites_SARS-CoV2). The A allele is one of the defining mutations of the Delta strain, and the apparent repeated re-emergence of the G allele within the Delta clade (Figure S1) is likely a consequence of this common sequencing error. Most other sites, including the site with the highest heterozygosity, position 23,604 (Figure 1C), do not show a similar pattern of homoplasy (Figure S2). This suggests that the sites with the highest apparent imputation error rate might in fact have a much lower true imputation error; the *tree imputation* method may provide a more accurate assignment of alleles than the reported sequencing data for some problematic sequencing sites.

### Simulations

**Figure 2:**
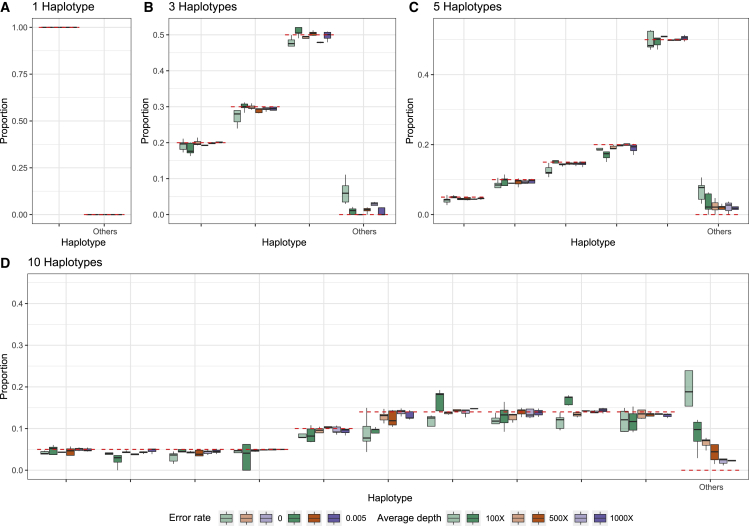
Estimated proportions for simulated 300 bp single-end reads Estimated proportions for simulated 300 bp single-end reads with five replicates for when the sample truly contains 1 (A), 3 (B), 5 (C), or 10 (D) haplotypes out of a total of 1,499,078 non-redundant candidate haplotypes in the database. The red dashed lines indicate the true proportion of each haplotype. "Other" indicates the sum of estimated proportions for all haplotypes that are not truly represented in the sample.

**Figure 3:**
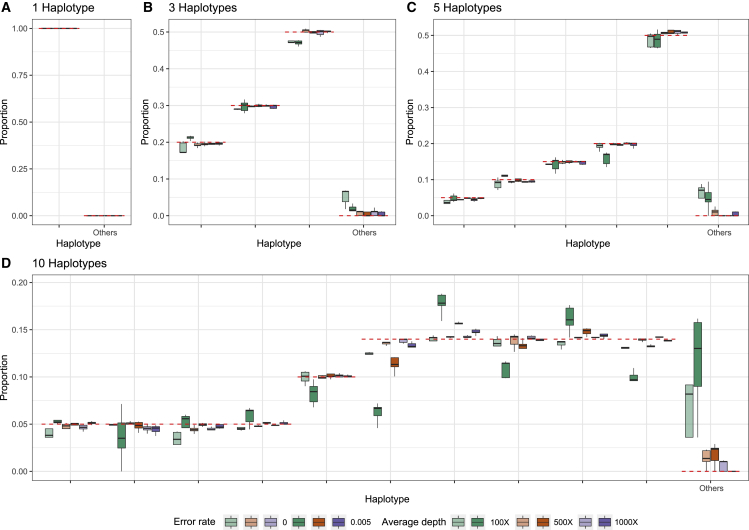
Estimated proportions for simulated paired-end reads (2 × 150 bp with an insert size of +25 bp) Estimated proportions for simulated paired-end reads (2 × 150 bp with an insert size of +25 bp) with five replicates for when the sample truly contains 1 (A), 3 (B), 5 (C), or 10 (D) haplotypes out of a total of 1,499,078 non-redundant candidate haplotypes in the database. The red dashed lines indicate the true proportion of each haplotype. “Other” indicates the sum of estimated proportions for all haplotypes that are not truly represented in the sample.

**Figure 4:**
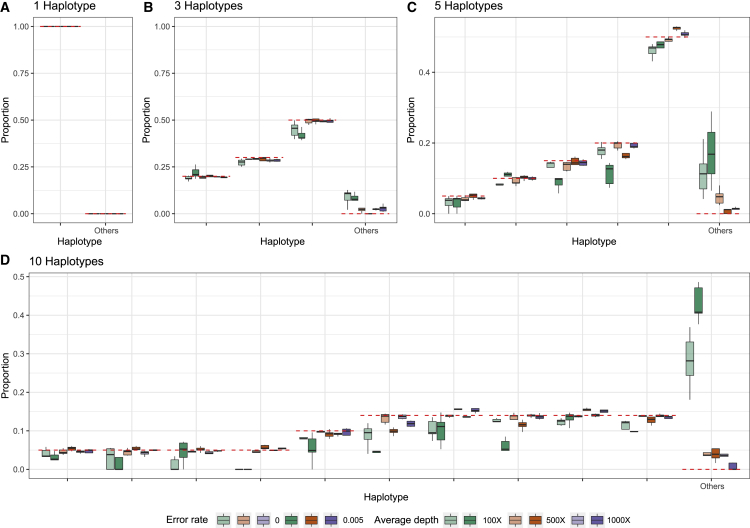
Estimated proportions for simulated paired-end reads (2 × 75 bp with an insert size of +25 bp) Estimated proportions for simulated paired-end reads (2 × 75 bp with an insert size of +25 bp) with five replicates for when the sample truly contains 1 (A), 3 (B), 5 (C), or 10 (D) haplotypes out of a total of 1,499,078 non-redundant candidate haplotypes in the database. The red dashed lines indicate the true proportion of each haplotype. “Other” indicates the sum of estimated proportions for all haplotypes that are not truly represented in the sample.

**Figure 5:**
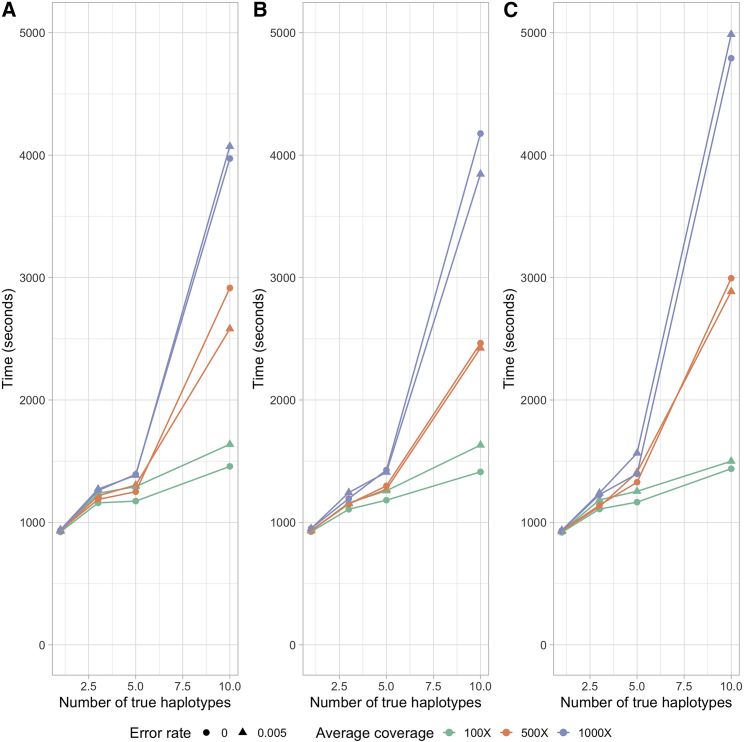
Average run times for read simulations Average run times for single-end 300 bp (A), paired-end 2 × 150 bp (B), and paired-end 2 × 75 bp (C) read simulations using 100×, 500×, and 1,000× average depth with an error rate of 0% and 0.5%. Each average run time reported is based on 5 replicates. Times were calculated using an AMD EPYC 7742 tetrahexaconta-core 2.25–3.40 GHz processor.

In the STAR Methods, we describe an algorithm for estimating the proportion of different SARS-CoV-2 haplotypes in an environmental sample using maximum likelihood. To evaluate the performance of the method, we simulate several sets of reads (single-end 300 bp, paired-end 2 × 150 bp, and paired-end 2 × 75 bp) from 1, 3, 5, and 10 haplotypes with average depths of 100×, 500×, and 1,000× and sequencing error rates of 0% and 0.5% (see STAR Methods). We then apply the method to these sets of reads using a database of 3,117,131 haplotypes, report the estimated proportions of each candidate haplotypes, and compare them with the truth (Figures 2, 3, and 4). In most cases, the estimates are close to the true proportions; however, with a low coverage and high error rate, the proportions of the true haplotypes will tend to be underestimated, and haplotypes that truly are not present will tend to be estimated as present in the sample. With one true haplotype in the sample, the proportion of this haplotype is always estimated to be 100%. For sufficiently high depth, e.g., 1,000× corresponding to roughly a total of 30 Mb data, the estimates of haplotype proportions are quite accurate, even when 10 haplotypes are present and for haplotypes with a proportion as low as 5%. There is similarly very little probability mass assigned to haplotypes that are not truly in the sample. For example, for 150 bp paired-end reads with a +25 bp insert and 1,000× average sequencing depth, the estimate of the cumulative average proportion of all haplotypes not truly in the sample is 0.63%. The speed of the method is highly dependent on the number of true haplotypes and the average depth (Figure 5), but for realistically sized datasets with a reference database of 3,117,131 haplotypes, the typical computational time is between 15 min and 2 h using a single core. This includes the initial time cost of ∼10.5 min for reading the large panel of reference haplotypes into memory. There is no appreciable difference in speed between the different sequencing strategies used, except that paired-end 2 × 75 bp sequences tends to take longer at higher average coverage. Simulations using the higher error rate (0.5%) are slower than simulations with no error. The average time for all sets of simulations with 5 or fewer true haplotypes is <30 min for all coverages, while the average time for 10 true haplotypes varies between ∼24 to ∼83 min depending on the average depth. Additionally, utilization of multiple cores during the creation of the mismatch matrix offers a substantial reduction in time (Figure S3). In order to quantify the statistical evidence for the presence of a candidate haplotype in the sample, we propose a likelihood ratio (LLR) test, formed by comparing the maximum likelihood value calculated when the candidate haplotype is eliminated from the sample (p = 0) with the maximum likelihood value calculated when allowing the haplotype to be present in the sample (p ≥ 0), where p is the proportion of the haplotype in the sample (see STAR Methods). Standard asymptotic theory for the distribution of the LLR statistics does not apply to this situation for several reasons: most importantly, a search is first made to find the haplotypes that provide the largest increase in the likelihood among many haplotypes, and we only calculate the LLR for the haplotypes with estimates of p > 0. We, therefore, use simulations to evaluate the distribution of the LLR test statistics under varying conditions. We simulated 1,000 datasets with different numbers of true haplotypes, coverage, read length, and error rate and calculated the LLR for all haplotypes that were falsely inferred to be present in the sample (Figure S4). Since the frequency of LLR >2 and LLR >4 is about 0.001 and 0.0005, respectively, we recommend using 2 and 4 as thresholds for strong and extremely strong evidence for presence of the haplotype in the sample.

### Application to wastewater data

**Figure 6:**
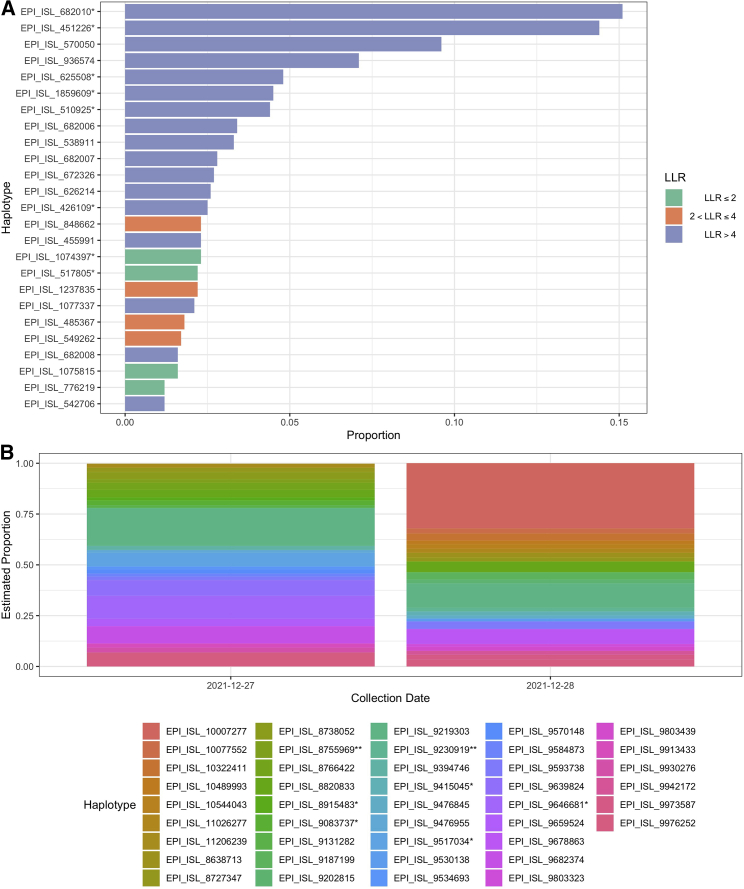
Estimated proportions of the top 25 haplotypes estimated from wastewater shotgun sequencing data (A) Estimated proportions of the top 25 haplotypes estimated from wastewater shotgun sequencing data from Crits-Christoph et al. (2021) and their log likelihood ratios. Haplotypes with an asterisk (∗) are identical to other haplotypes. EPI_ISL_682010∗ is identical to EPI_ISL_682025, EPI_ISL_1373628, EPI_ISL_1373632, and EPI_ISL_1373659. EPI_ISL_451226∗ is identical to EPI_ISL_451227 and EPI_ISL_455983. EPI_ISL_625508∗ is identical to EPI_ISL_625520, EPI_ISL_672318, EPI_ISL_672449, EPI_ISL_739003, EPI_ISL_739029, EPI_ISL_739135, EPI_ISL_739161, EPI_ISL_739207, and EPI_ISL_739286. EPI_ISL_1859609∗ is identical to EPI_ISL_1859762. EPI_ISL_510925∗ is identical to EPI_ISL_510926. EPI_ISL_426109∗ is identical to EPI_ISL_486012, EPI_ISL_570168, EPI_ISL_570172, EPI_ISL_576500, and EPI_ISL_576501. EPI_ISL_1074397∗ is identical to EPI_ISL_2190584. EPI_ISL_517805∗ is identical to EPI_ISL_527398 and EPI_ISL_137362. (B) Estimated proportions of haplotypes from wastewater samples collected from Point Loma Wastewater Treatment Plant in San Diego, CA, on December 27 and 28, 2021. These samples correspond to SRA: SRR18541028 and SRA: SRR18541040, respectively, in the NCBI Sequence Read Archive (SRA). An asterisk (∗) denotes haplotypes that are identical, and two asterisks (∗∗) denote haplotypes that are in an unidentifiable group. EPI_ISL_9517034∗ is identical to EPI_ISL_9570257. EPI_ISL_9646681∗ is identical to EPI_ISL_9647084 and EPI_ISL_9647386. EPI_ISL_9415045∗ is identical to EPI_ISL_8772397, EPI_ISL_8573946, EPI_ISL_9461694, EPI_ISL_9499928, EPI_ISL_10739875, EPI_ISL_10739633, EPI_ISL_10175015, EPI_ISL_9125128, EPI_ISL_9461721, EPI_ISL_9467296, EPI_ISL_9515067, EPI_ISL_9515028, EPI_ISL_10739800, EPI_ISL_9395085, EPI_ISL_9614169, EPI_ISL_9614158, EPI_ISL_9614168, EPI_ISL_11140829, EPI_ISL_9735298, EPI_ISL_9735205, EPI_ISL_9735252, EPI_ISL_9735108, EPI_ISL_9791610, EPI_ISL_9850125, EPI_ISL_9908638, EPI_ISL_9753039, EPI_ISL_9964765, and EPI_ISL_10717937. EPI_ISL_9083737∗ is identical to EPI_ISL_9218282. EPI_ISL_8915483∗ is identical to EPI_ISL_9279835. EPI_ISL_8755969∗∗ is in an unidentifiable group with identical haplotypes EPI_ISL_9277900 and EPI_ISL_10842235. Identical haplotypes EPI_ISL_9230919∗∗ and EPI_ISL_9057497 are in an unidentifiable group with identical haplotypes EPI_ISL_9220378, EPI_ISL_9220183, EPI_ISL_8859542, EPI_ISL_8837776, and EPI_ISL_9017371.

**Figure 7:**
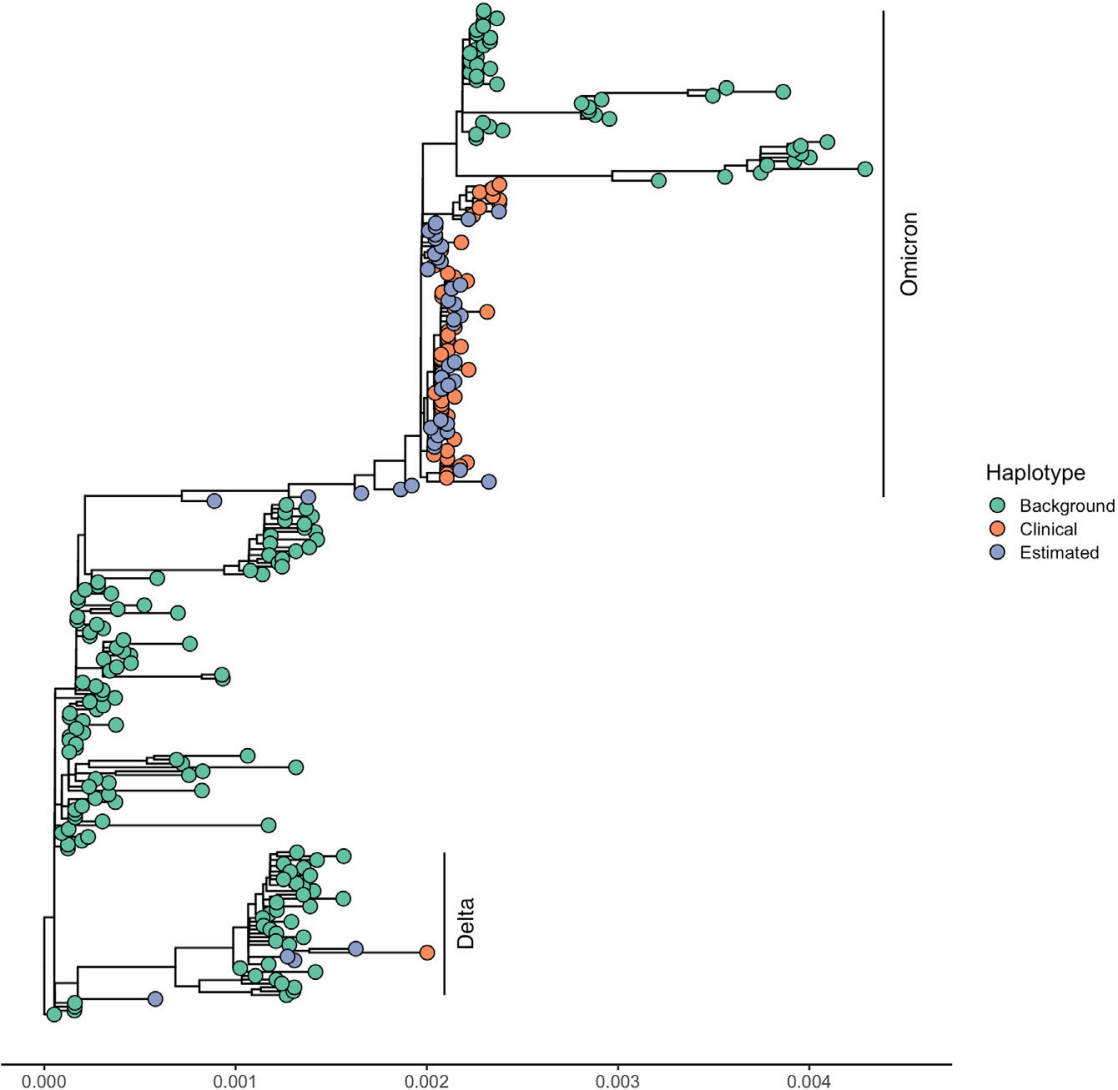
Estimated maximum likelihood phylogenetic tree of 261 SARS-CoV-2 sequences, which includes 173 background sequences (green nodes), 44 sequences estimated by the method (purple nodes), and 44 randomly chosen clinical sequences from San Diego County from January 3 and 4, 2022 (red nodes) The x axis is the number of substitutions per site. The Omicron and Delta clades are labeled. The maximum likelihood estimate of the phylogeny was obtained using the program RAxML under the GTR + Γ model of DNA substitution (Stamatakis, 2014). The multiple sequence alignment was created using FAMSA (Deorowicz et al., 2016) with default settings. Table S1 lists the sequences used in this analysis.

To apply our method to a published dataset, we estimated the composition of SARS-CoV-2 haplotypes using wastewater shotgun sequencing data from Crits-Christoph et al. (2021) in Figure 6A and from Karthikeyan et al. (2021) in Figure 6B. The data from Crits-Christoph et al. (2021) was all collected in the San Francisco Bay Area. Two out of the top ten haplotypes were collected in Alameda County (EPI_ISL_625508, which is identical to EPI_ISL_625520, and EPI_ISL_672326), and the top five haplotypes were all collected in North America. The data from Karthikeyan et al. (2021) were collected at the Point Loma Wastewater Treatment Plant in San Diego, CA, on December 27 and 28, 2021. We identified an increasing proportion for EPI_ISL_9593738 (0.017–0.038) and decreasing proportions for EPI_ISL_9976252 (0.069–0.032) and EPI_ISL_8727347 (0.022–0.019). EPI_ISL_9593738, EPI_ISL_9976252, and EPI_ISL_8727347 are all designated as BA.1.1 (Pango v.4.0.6 PLEARN-v.1.8). The estimated haplotypes are representative of the variability of circulating strains and are nearly identical to the clinical samples that were collected in San Diego County and were deposited to GISAID 1 week after the Point Loma wastewater sample collection dates. We illustrate this by estimating a phylogenetic tree of the clinical samples and the estimated samples on a background set of SARS-CoV-2 genomes (Figure 7). The clinical samples and the estimated samples cluster together in two clades. The Omicron clade contains most of the clinical and estimated wastewater haplotypes, with little divergence among the sequences and with wastewater inferred haplotypes and clinical haplotypes clustering among each other. A second set of wastewater inferred sequences cluster with the Delta clade containing the remainder of the clinical and estimated samples. Furthermore, the average genetic distance between clinical and estimated haplotypes, 3.99 × 10^−4^, is very similar to the average distance among the estimated haplotypes, 4.45 × 10^−4^. The clinical samples had an average genetic distance of 3.05 × 10^−4^.

## Discussion

In order to allow for accurate inferences of haplotype composition, we first developed a new phylogenetic method for data imputation for SARS-CoV-2 sequences. The method proved to be highly accurate with error rates comparable to, or lower than, typical sequencing error rates (Figure 1A). In fact, apparent wrongly inferred nucleotides may, in many cases, not be wrongly inferred but rather be inferences of the true allele, correcting a sequencing error in the reported sequence. Thus, similarly to imputation-based genotype calling in humans, this method could be used for correcting sequencing errors and could be incorporated formally into an algorithm of imputation-informed sequencing where the quality scores from sequencing reads are combined with phylogenetically informed nucleotide probabilities to call nucleotides in each position. Computationally, this could be done simply by using the phylogenetic posterior probabilities of nucleotides as priors for genotype calling. Our simulation results for the EM algorithm show that the new method can accurately estimate proportions of SARS-CoV-2 lineages in wastewater samples when up to 10 haplotypes with frequencies as low as 5% are represented in the sample. Nonetheless, the estimated proportions for the true haplotypes tend to be slightly lower than the actual proportions because the presence of other non-true haplotypes is also estimated at a low frequency. In order to have some probability for other non-true haplotypes to be estimated, the true proportions for the true haplotypes will naturally, on average, be slightly underestimated. In all sets of simulations of single-end 300 bp (Figure 2), paired-end 2 × 75 bp (Figure 3), and paired-end 2 × 150 bp reads (Figure 4), the estimated proportions of the true haplotypes tend to be more accurate as sequencing depth increases. When there are many haplotypes (i.e., when there are 10 true haplotypes) and sequencing depth is low (i.e., 100×), there is a high degree of noise in the dataset. However, as the total sequencing depth increases, the estimates become progressively more accurate. We recommend that studies focused on identifying different haplotypes of SARS-CoV-2 in environmental samples aim to achieve an average depth of 1,000×. Additionally, the method presented here has only been evaluated for the estimation of proportions of haplotypes with a frequency of 5% or larger. We recommend that haplotypes identified in the sample at low frequencies are evaluated using the LLR test, as they likely could be false positives.

### Conclusions

Current strategies for monitoring community composition of SARS-CoV-2 haplotypes include sequencing a large number of clinical samples. As SARS-CoV-2 becomes endemic, tracking the relative prevalence in local communities of different SARS-CoV-2 haplotypes will be highly costly. Furthermore, the use of clinical samples is associated with a lag from infection onset to hospitalization. Our results suggest an alternative strategy of monitoring using wastewater samples. Estimating relative proportions of haplotypes also directly allows for the estimation of relative proportions of lineages, as each haplotype can be assigned to a Pangolin lineage (O’Toole et al., 2021). Wastewater sequencing has already proved effective for tracking SARS-CoV-2 abundance (Korber et al., 2020; Rockett et al., 2020). With the computational framework developed here, it also promises to become an important cost-effective strategy for monitoring the local composition of different viral haplotypes.

### Limitations of the study

A central limitation of the method is that it requires the availability of a comprehensive and well-curated database. Haplotypes not present in the database cannot be identified using this method. Another limitation relates to frequency threshold (i.e., <1% allele frequency threshold), which is used to filter out unlikely haplotypes among the millions of possible haplotypes. If a true haplotype is present in the sample in a proportion less than the allelic frequency threshold, our method would not be able to detect the true haplotype. The number of true haplotypes that the method is able to detect is also a limitation. We have here assumed that there are between 1 and 10 haplotypes in each sample. If there are substantially more haplotypes than this, the method is not expected to be able to accurately identify the proportions of all haplotypes. While the inference of haplotypes does not rely on a tree, the inference of missing data in the reference data does. A tree is commonly assumed for most evolutionary analyses of SARS-CoV-2, such as common phylodynamic analyses (Duchene et al., 2020; Dellicour et al., 2021). Nonetheless, recombination is common among coronaviruses (Müller et al., 2021), and it is therefore relevant to consider the possibility of recombination playing a role in SARS-CoV-2 as well. In fact, there have been several reported observations of recombination in SARS-CoV-2 (Wertheim et al., 2022; Ignatieva et al., 2022). A heuristic approach to this problem might be to simply remove apparent recombinant sequences, but if recombination is frequent, this approach may not be feasible. An alternative approach would be to estimate local trees in the genome. Such an approach can readily be adapted in the framework proposed here for inference of missing data.

## STAR★Methods

### Key resources table

**Table:** 

REAGENT or RESOURCE	SOURCE	IDENTIFIER
**Deposited data**		

Bay Area Wastewater Data	Crits-Christoph et al. (2021)	NCBI BioProject: PRJNA661613
Point Loma Wastewater Data	Karthikeyan et al. (2021)	NCBI BioProject: PRJNA819090
Simulated data	This paper	Zenodo: https://doi.org/10.5281/zenodo.5838942
Imputed MSA	This paper	Zenodo: https://doi.org/10.5281/zenodo.5838946
Sequences used in Figure 7	This paper	Mendeley Data: https://doi.org/10.17632/j9pdn88sx5.1

**Software and Algorithms**		

Estimation method	This paper	Zenodo: https://doi.org/10.5281/zenodo.6999441

### Resource availability

#### Lead contact

Further information and requests should be directed to and will be fulfilled by the lead contact, Dr. Lenore Pipes (lpipes@berkeley.edu).

#### Materials availability

This study did not generate any new materials.

### Method details

#### SARS-CoV-2 reference database

To build the SARS-CoV-2 reference database, a multiple sequence alignment (MSA) of 3,117,131 SARS-CoV-2 genomes (msa_2021-10-15.tar.xz) and the corresponding phylogenetic tree (GISAID-hCoV-19-phylogeny-2021-10-13.zip) was downloaded from GISAID (www.gisaid.org) on October 16, 2021. We pruned sequence EPI_ISL_4989640 from the tree since it was not present in the MSA. We use the function collapse.singles to collapse elbow nodes (i.e., nodes other than the root with two degrees) and multi2di to resolve multichotomies in the R ape package (Paradis et al., 2004). We impute missing data (i.e., every position in the MSA that did not contain an A, G, C, or T), using the phylogenetic tree. To do so, we first scale the branch lengths in terms of substitutions per site by dividing each reported branch length by the average sequence length (29618.5). For branch lengths that were reported to be 0, we define them to be 0.01 divided by the average sequence length. We impute missing nucleotides using the maximum of the posterior probability of each nucleotide in the leaf nodes under a standard Jukes and Cantor model (Jukes and Cantor, 1969), using standard computational algorithms (Yang, 2014). In brief, because the model is time-reversible, the root can be placed in any particular node, and the fractional likelihoods (joint probabilities of a fraction of the data in the leaf nodes and the nucleotide state in the node) can be pulled recursively towards the node from both the child nodes and the parental node. The posterior probability in the leaf nodes of a nucleotide is calculated as the product of the stationary probability of the nucleotide multiplied by the fractional likelihood in the leaf node conditioned on the data in all other leaf nodes. This can be programmed so the calculation is linear in the number of leaf nodes using a single pre-order and a single post-order traversal of the tree that will calculate the posterior probabilities in all nodes. We note that other models than the Jukes and Cantor model could provide more accurate estimates, but at a computational cost. Since calculating fractional likelihoods for the entire tree requires more RAM than was computationally feasible for us ( ∼ 72TB of RAM), we split the tree into partitions, and process each partition sequentially as follows: Each internal node in the tree corresponds to a partition of leaf nodes into three sets. First, we identify the node with the minimum variance in the number of elements among these three partitions, i.e. we find

(Equation 1) $$\underset{n\in T}{\text{min}}\left(\frac{{\left(n_{a}-\frac{n_{1}+n_{2}+n_{a}}{3}\right)}^{2}+{\left(n_{1}-\frac{n_{1}+n_{2}+n_{a}}{3}\right)}^{2}+{\left(n_{2}-\frac{n_{1}+n_{2}+n_{a}}{3}\right)}^{2}}{3}\right)$$

where *n* is a node in the tree, *T* is the tree, $n_{1}$ is the number of leaf nodes descending from the left child of *n*, $n_{2}$ is the number of leaf nodes descending from the right child, and $n_{a}=N-n_{1}-n_{2}$, where *N* is the total number of leaf nodes in the tree. We then split the tree into 3 subtrees by eliminating the identified node. We then iterate this procedure for the resulting subtrees until all trees contain at most 50,000 leaf nodes. Using this partitioning procedure, we obtain 121 trees which we use to calculate the posterior probabilities at each site. After imputation, we trim the MSA to begin at the start of the Wuhan reference sequence (Wuhan-Hu-1), position 55 in the MSA, and we removed every position in the MSA that contains a gap in Wuhan-Hu-1. After this trimming and imputation process, we save non-informative invariant sites (856 sites), in order to reduce running time when eliminating unlikely haplotypes. We also remove all identical sequences, resulting in 1,499,078 non-redundant genomes.

#### Estimating the proportions of SARS-CoV-2 genomes

All sequencing reads are aligned to Wuhan-Hu-1 (NC_045512.2) using bowtie2 (Langmead and Salzberg, 2012) with the following command for single-end reads, bowtie2 --all -f -x wuhCor1 -U, and for paired-end reads, bowtie2 --all -f -x wuhCor1 -1 -2. For each read data set, we first remove unlikely genomes from the candidate haplotype alignment by eliminating genomes with SNP alleles that have an allele frequency in the read data less than a user-defined frequency threshold. For the analyses in this data, that threshold was set to 0.01. This typically reduced the size of the alignment to <1,000 relevant genomes. Using this reduced set of SARS-CoV-2 genomes, we calculate a matrix of dimensions (number of reads) × (number of genomes) containing the number of mismatches between each sequencing read and each genome, $d=\left\{d_{ij}\right\}$. For paired-end reads with reads that overlap, we use the consensus nuleotide. If there is a conflict at any position in the overlap of the paired-end reads, we omit this site. Based on the mismatch matrix, *d*, we first calculate the probability of observing read *j* given that it comes from haplotype *i*, denoted as $q_{ij}$. Assuming that the reads are independent (PCR clones removed) and a user-defined error rate α (default = 0.005) at each nucleotide, this probability is given by

$$q_{ij}=\alpha^{d_{ij}}\times {\left(1-\alpha \right)}^{n_{j}-d_{ij}}$$

where $n_{j}$ is the length of read *j* and $d_{ij}$ is the number of mismatches in read *j* given that it comes from haplotype *i*. The log-likelihood is then given by

(Equation 2) $$\text{log}L\left(p_{1}\text{,}\;\cdots \text{,}\;p_{k}\right)=\sum_{\text{j}=1}^{\text{n}}\text{log}\sum_{i=1}^{k}q_{ij}p_{i}\text{,}$$

where $p_{i}$ (*i* = 1,⋯,k) is the proportion of haplotype *i*, i.e. the parameters we wish to estimate. We then use the standard Expectation Maximization (EM) algorithm (Dempster et al., 1977) to maximize the likelihood function with respect to these parameters (Algorithm 1):

Algorithm 1 **EM algorithm for estimating the proportions of candidate haplotypes**

**Input**: The probability of observing read *j* given that it comes from haplotype *i*, $q_{ij}$, for all *i* and *j*.

**Output**: The proportion of each candidate haplotype, $p_{i}$, for all *i*.

1. Initialize the proportions of each haplotype $p_{i}\left(0\right),i=1\ldots k$, with uniform probabilities U(0,1) and then re-scaled to 1.
2. Compute the log-likelihood $\ell_{0}=\sum_{\text{j}=1}^{\text{n}}\text{log}\sum_{i=1}^{k}q_{ij}p_{i}\left(0\right)$;
3. repeat
4. Compute the proportion of each candidate haplotype at iteration *t* as $p_{i}\left(t\right)=\frac{1}{n}\sum_{j=1}^{n}\frac{p_{i}\left(t-1\right)q_{ij}}{\sum_{l=1}^{k}p_{l}\left(t-1\right)q_{lj}}$;
5. Compute the log-likelihood at iteration *t* as $\ell_{t}=\sum_{\text{j}=1}^{\text{n}}\text{log}\sum_{i=1}^{k}q_{ij}p_{i}\left(t\right)$;
6. until $\ell_{t}-\ell_{t-1}<\varepsilon$, where ε is a pre-defined stopping criterion.

However, Algorithm 1 usually has a slow convergence rate, especially when the number of candidate haplotypes *k* is large. Therefore, to accelerate the Algorithm 1, we use the SQUAREM algorithm proposed by Varadhan and Roland (2008) with its implementation in the R package turboEM (Bobb and Varadhan, 2020).

#### Determining unidentifiable haplotypes

Note that if two haplotypes have the same $q_{ij}$’s, say there exist *i* and $i^{\text{′}}$ such that $q_{ij}=q_{i^{\text{′}}j}$ for all j=1,⋯,n, the log-likelihood (2) becomes

(Equation 3) $$\text{log}\;L=\sum_{j=1}^{n}\text{log}\left[\left(\underset{r\in \left\{1\text{,}\;\ldots \;\text{,}k\right\}/\left\{i\text{,}i^{\text{′}}\right\}}{\sum }q_{rj}p_{r}\right)+q_{ij}\left(p_{i}+p_{i^{\text{′}}}\right)\right]\text{.}$$

Therefore, as long as $p_{i}+p_{i^{\text{′}}}$ is fixed, (3) remains the same no matter what value $p_{i}$ and $p_{i^{\text{′}}}$ take, making the model unidentifiable. To solve this problem, we gather haplotypes with the same ${\left\{q_{ij}\right\}}_{j=1}^{n}$ into an unidentifiable group and estimate its overall proportion instead of the proportions of each haplotype in it.

#### Quantifying the statistical evidence of the existence of each candidate haplotype

To provide a measure of statistical support for the presence of haplotype $i_{0}$, i.e. $p_{i_{0}}>0$, we remove haplotype $i_{0}$ from the candidate set of haplotypes and re-run Algorithm 1 providing a new estimate ${\left\{{\tilde{p}}_{i}\right\}}_{i=1}^{k}$ with ${\tilde{p}}_{i_{0}}=0$. Using Equation (2), we can then calculate the difference in log likelihood before and after removing haplotype $i_{0}$, denoted as ${\text{LLR}}_{i_{0}}$. From our simulations (see results), we recommend using ${\text{LLR}}_{i_{0}}\geq 4$ as strong statistical evidence in favor of existence of haplotype $i_{0}$ in the sample.

#### Simulating missing data for imputation

For every SARS-CoV-2 genome (out of a total of 3,117,131 genomes), we randomly remove 1% of nucleotides, and save the true nucleotide at each position that was removed. We then use the *Tree imputation* method and the *Common allele* method to impute the nucleotides that are missing.

#### Simulating reads from SARS-CoV-2 genomes

We choose 10 haplotypes among 1,499,078 haplotypes uniformly at random. Then, to simulate single-end reads from a haplotype, we choose a starting point uniformly at random and let it extend $m_{0}$ bps, where $m_{0}$ is the read length. For paired-end reads, we similarly choose a starting point at random and let it extend $m_{0}$ bps. Then, starting from the end of this read, if the insert size is $m_{1}$ is positive, we simulate the start of the reverse read $m_{1}$ bps forward with length $m_{0}$; if $m_{1}$ is negative, we simulate the start of the reverse read $m_{1}$ bps backwards. We then add sequencing errors independently with probability α=0.005 at each site. Errors are induced by relabeling the nucleotide to any of the other three possible nucleotides with the following probabilities used in Stephens et al. (2016):

$$\begin{array}{ll} \; & \begin{array}{cccc} \;A\; & \;C\; & \;G\; & \;T\; \end{array}\\ \begin{array}{l} A\\ C\\ G\\ T \end{array} & \left[\begin{array}{llll} \;0\; & 0.4918 & 0.3377 & 0.1705\\ 0.5238 & \;0\; & 0.2661 & 0.2101\\ 0.3754 & 0.2355 & \;0\; & 0.3890\\ 0.2505 & 0.2552 & 0.4942 & \;0\; \end{array}\right] \end{array}$$

#### Calculating time cost

To calculate running time of the method we use /usr/bin/time on an AMD EPYC 7742 tetrahexaconta-core 2.25–3.40 GHz processor and report real time in the results (Figure 5). The running time that we calculate includes running the method from start (reading in the reference haplotypes) to finish (reporting proportions) and includes the filtering step for eliminating unlikely haplotypes. We report times that do not include calculating the log-likelihood ratio.

#### Applying the method to wastewater data from Crits-Christoph et al. (2021) and Karthikeyan et al. (2021)

Wastewater shotgun sequencing data from Crits-Christoph et al. (2021) was downloaded from NCBI BioProject: PRJNA661613 (https://www.ncbi.nlm.nih.gov/bioproject/PRJNA661613). All samples were pooled together and aligned against Wuhan-Hu-1 using BWA-MEM (Li, 2013) to identify SARS-CoV-2 reads. Wastewater shotgun sequencing data from Karthikeyan et al. (2021) was downloaded from NCBI BioProject: PRJNA819090 (https://www.ncbi.nlm.nih.gov/bioproject/PRJNA819090) and samples SRA: SAMN27108230, and SRA: SAMN27108220 (https://www.ncbi.nlm.nih.gov/biosample/SAMN27108230 and https://www.ncbi.nlm.nih.gov/biosample/SAMN27108220, respectively). Samples from Karthikeyan et al. (2021) were analyzed using a reference database created from a GISAID SARS-CoV-2 global phylogeny from March 21, 2022 that was filtered for sequences from January 1, 2022 to January 31, 2022. For genetic distances, we calculate genetic distance using dnadist using the F84 model from the PHYLIP package.

### Quantification and statistical analysis

All details regarding the method are fully explained in the section method details. Here we provide a brief summary of certain analyses, parameters, and software that were used. The section of method details entitled ”Estimating the proportions of SARS-CoV-2 genomes” uses statistical analyses using base R v.4.0.5. To implement the SQUAREM algorithm, we use turboEM v.2020.1 (Bobb and Varadhan, 2020). We use the "parameter" type of convergence criterion with tolerance $10^{-7}$. In the section of method details entitled ”Quantifying the statistical evidence of the existence of each candidate haplotype”, to quantify the statistical evidence for the presence of a candidate haplotype in the sample, we use empirical thresholds, which correspond to p-value <0.001 and <0.0005, respectively. In the section of method details entitled “Simulating missing data for imputation”, to remove missing nucleotides at random for our imputation method, we use the perl function rand(). To evaluate the performance of our method in Figure 2, Figure 3, Figure 4 and estimate its running speed in Figure S3, we use five replicates. To estimate the phylogenetic tree in Figure 7, we use mafft --maxiterate 1000 --globalpair for the alignment and raxmlHPC-PTHREADS -m GTRGAMMA -p 1234 to estimate the tree.

## Data Availability

All original code, simulated data, and the imputed MSA has been deposited to Zenodo and is publicly available as of the date of publication. DOIs are listed in the key resources table. Additional Supplemental Items are available from Mendeley Data: https://doi.org/10.17632/j9pdn88sx5.1.

## Supplemental information

Document S1. Figures S1–S4

Table S1. List of background, clinical, and estimated SARS-CoV-2 sequences used to estimate phylogenetic tree, related to Figure 7

Document S2. Article plus supplemental information
