# Supplementary material for: Estimating the relative proportions of SARS-CoV-2 haplotypes from wastewater samples
Source: Cell Rep Methods. 2022 Sep 20;2(10):100313. doi: 10.1016/j.crmeth.2022.100313 (PMC9485417; doi:10.1016/j.crmeth.2022.100313)
Supplement: Document S1. Figures S1–S4 [file mmc1.pdf]

Cell Reports Methods, Volume *2*

Supplemental information

Estimating the relative proportions of SARS-CoV-2 haplotypes from wastewater samples

Lenore Pipes, Zihao Chen, Svetlana Afanaseva, and Rasmus Nielsen

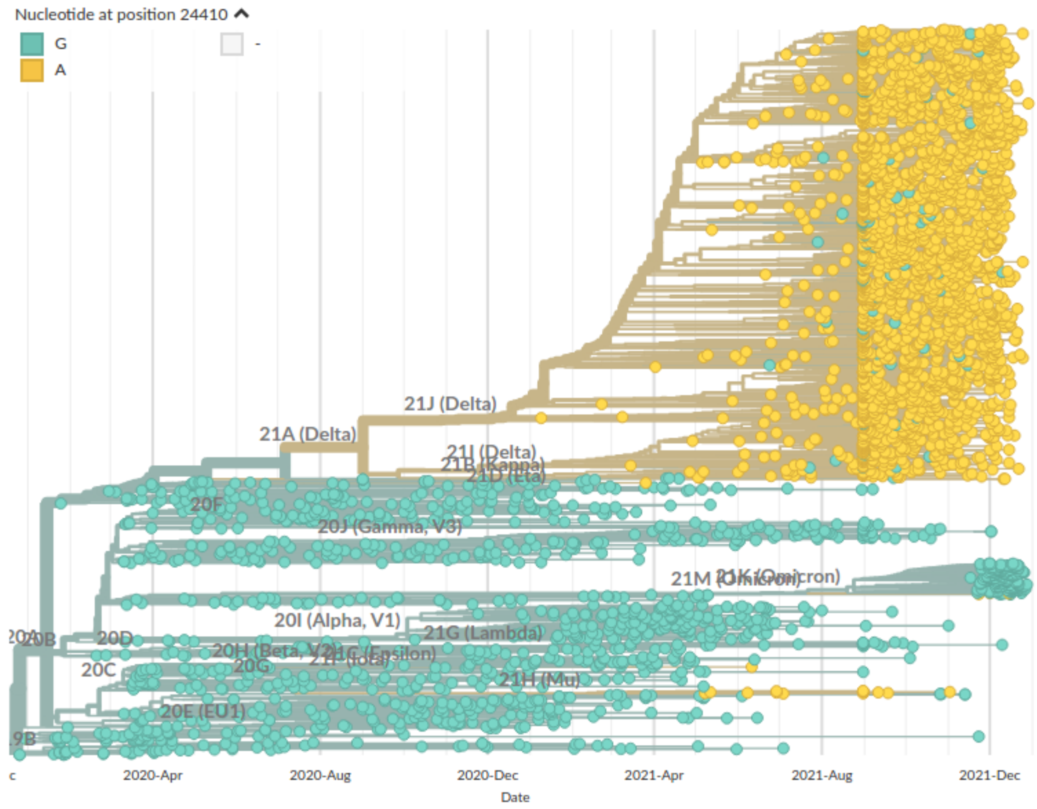

Figure S1: Screenshot of SARS-CoV-2 phylogeny from nextstrain.org for nucleotide position 24,410 taken on January 5, 2022. Related to Figure 1.

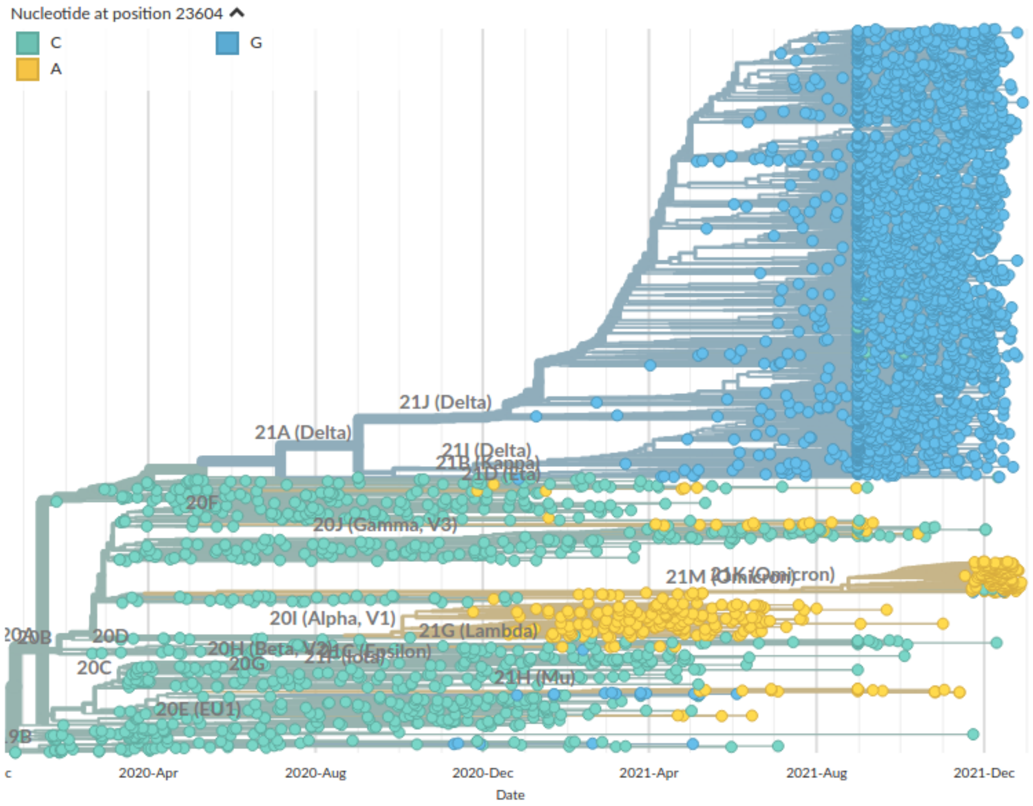

Figure S2: Screenshot of SARS-CoV-2 phylogeny from nextstrain.org for nucleotide position 23,604 taken on January 5, 2022. Related to Figure 1.

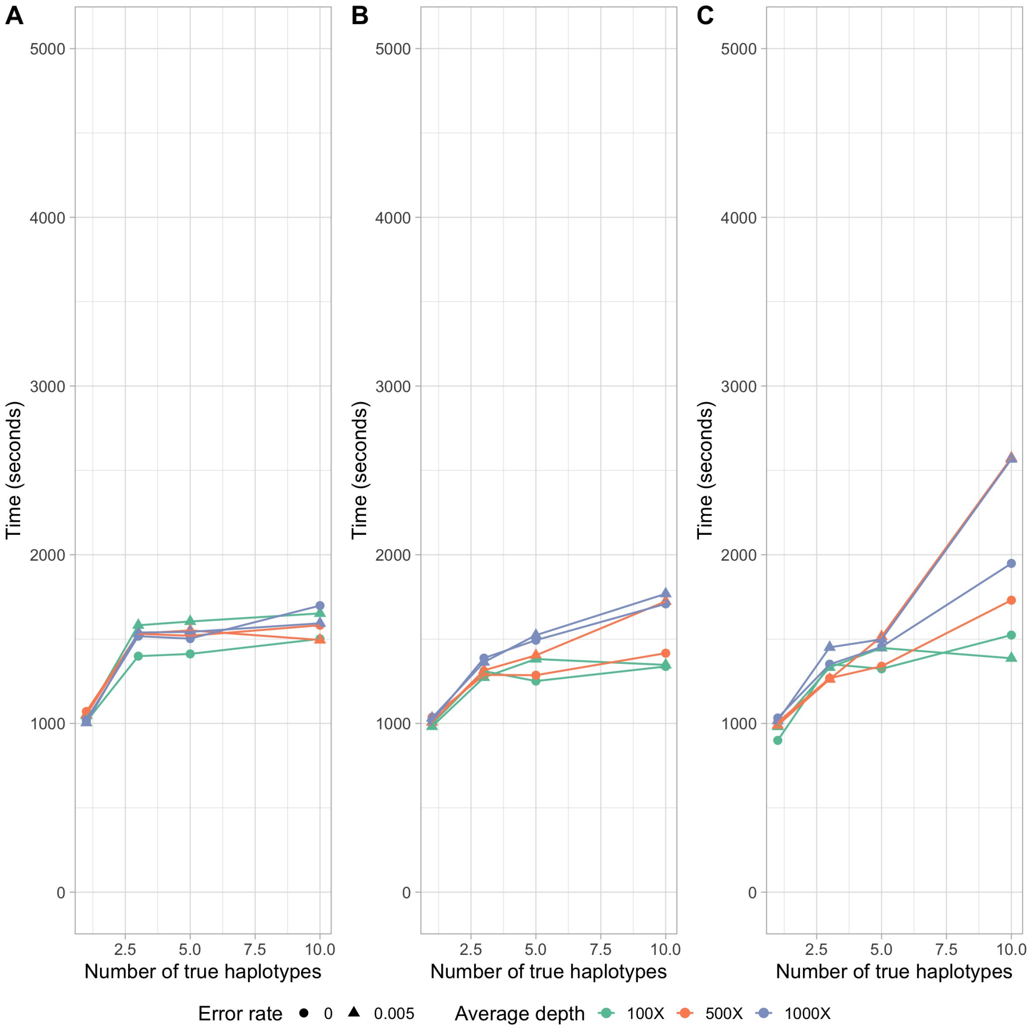

Figure S3: Average run times for utilization of multiple cores on single-end 300 bp (A), paired-end 2x150 bp (B), and paired-end 2x75bp (C) read simulations using 100X, 500X, and 1000X average depth with an error rate of 0% and 0.5%. 24 cores were used to for printing the mismatch matrix. Each average run time reported is based on 5 replicates. Related to Figure 5.

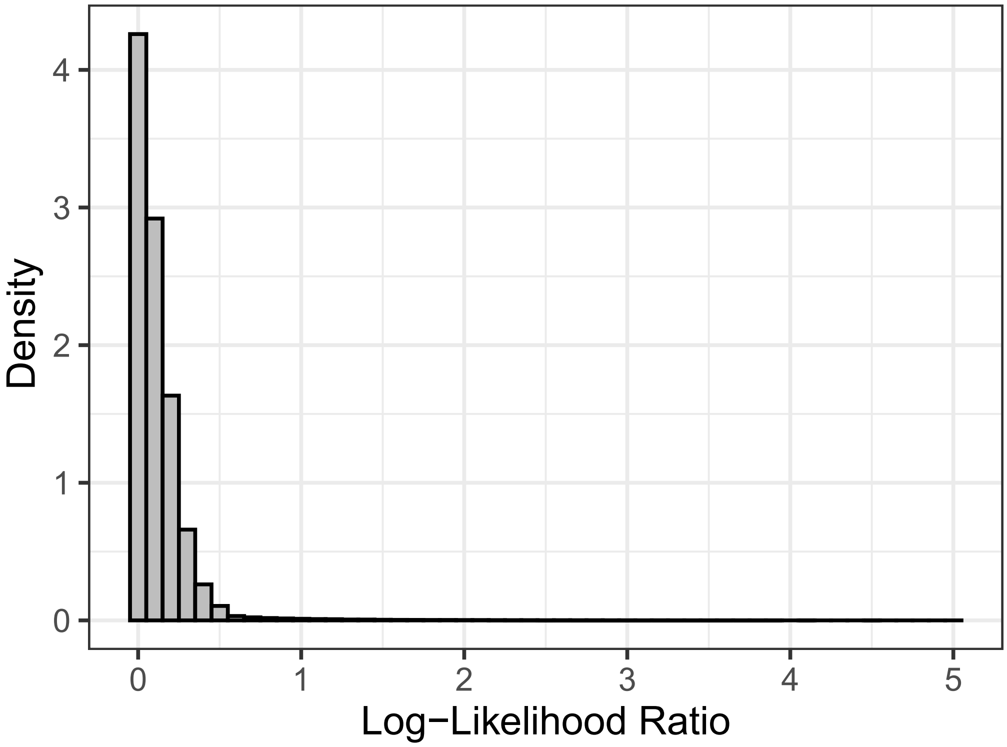

Figure S4: Distribution of log-likelihood ratios from 1,000 data sets of simulated $100 \sim 300bp$ single-end reads. Those simulated data sets include $3 \sim 10$ haplotypes with proportions ranging from 5% to 50% and average depths ranging from 50X to 300X. The error rate varies from 0% to 0.5%. Related to "Quantifying the statistical evidence of the existence of each candidate haplotype" in the STAR Methods.
